# Acidic Polysaccharide from *Angelica sinensis* Reverses Anemia of Chronic Disease Involving the Suppression of Inflammatory Hepcidin and NF-*κ*B Activation

**DOI:** 10.1155/2017/7601592

**Published:** 2017-09-24

**Authors:** Kaiping Wang, Jun Wu, Fang Cheng, Xiao Huang, Fang Zeng, Yu Zhang

**Affiliations:** ^1^Hubei Key Laboratory of Natural Medicinal Chemistry and Resource Evaluation, Tongji Medical College of Huazhong University of Science and Technology, No. 13, Hangkong Road, Wuhan 430030, China; ^2^Union Hospital of Tongji Medical College, Huazhong University of Science and Technology, No. 1277, Jiefang Road, Wuhan 430022, China

## Abstract

Anemia of chronic disease (ACD) is the second most prevalent anemia and frequently occurs in patients with acute or chronic immune activation. In the current study, we evaluated the therapeutic efficacy of *Angelica sinensis* polysaccharide (ASP) against ACD in rats and the potential mechanisms involved. The results showed that ASP inhibited inflammatory hepcidin in both HepG2 cells and ACD rats by blocking the IL-6/STAT3 and BMP/SMAD pathways. In ACD rats, the administration of ASP increased ferroportin expression, mobilized iron from the liver and spleen, increased serum iron levels, caused an elevation of serum EPO, and effectively relieved the anemia. Furthermore, ASP inhibited NF-*κ*B p65 activation via the I*κ*B kinases- (IKKs-) I*κ*B*α* pathway, thereby reducing the secretion of interleukin-6 (IL-6) and TNF-*α*, which is known to inhibit erythropoiesis. Our findings indicate that ASP is a potential treatment option for patients suffering from ACD.

## 1. Introduction

Anemia of chronic disease (ACD, or anemia of inflammation) is an acquired disorder and occurs simultaneously with acute or chronic immune activation, such as infection, autoimmune disorders, and cancer [1]. The dysregulation of iron homeostasis, associated with iron sequestration within the cells of the reticuloendothelial system (RES) and the limited availability of iron for erythropoiesis, is a major pathophysiological mechanism underlying ACD [2]. This iron sequestration is primarily mediated by hepcidin [3], the central regulator of iron homeostasis, which is stimulated by inflammation as a host defense against invading pathogens. In addition to stimulating a hepcidin increase, enhanced inflammation and cytokine activity directly suppress erythropoiesis and shorten erythrocyte survival, which further contribute to the development of ACD. Multiple cytokines, including IFN-*α*, IFN-*β*, IFN-*γ*, TNF-*α*, and IL-1, are reported to suppress erythropoiesis by inducing the apoptosis of erythroid burst-forming units (BFU-e) and colony-forming units (CFU-e) [4]. Moreover, cytokine-inducible radicals, such as nitric oxide or superoxide anion [5], may directly inhibit the proliferation of erythroid progenitor cells. The formation of erythropoietin (EPO), as well as the responsiveness of erythroid progenitor cells to EPO, is negatively affected and modulated by inflammatory cytokines [6]. The shortened erythrocyte survival is attributed to the erythrophagocytosis of macrophages activated by inflammatory cytokines [7].

The development of anemia impairs a patient's performance, quality of life, or prognosis of other underlying chronic diseases, and thus, direct treatment of anemia is considered when treating the underlying disease is not feasible. Blood transfusions, erythropoiesis-stimulating agents (ESAs), and intravenous iron have been introduced for ACD treatment in clinical practice. Although, such therapies either have a limited success in patients or cause adverse effects, such as increased mortality, risk of infection, iron overload, or recurrence of cancer [1, 8]. Therefore, there is a need for highly efficient drugs with limited side effects for the treatment of ACD.

The root of *Angelica sinensis* (Oliv.) Diels (*A. sinensis*), also known as “Dang Gui” in China, has been used historically in prescriptions or functional foods for replenishing blood, treating gynecological diseases, and preventing inflammation [9]. Among the several substances responsible for these bioactivities, polysaccharides are well studied. *Angelica sinensis* polysaccharide (ASP), a water-soluble acidic polysaccharide, was isolated and purified from the root of *A. sinensis*. Our previous studies showed that ASP reduces serum hepcidin levels and regulates iron homeostasis in iron deficiency anemia rats [10, 11]. In addition, ASP exerts anti-inflammatory effects by inhibiting T lymphocyte proliferation in concanavalin A-induced liver injury in mice [12]. However, the effects of ASP and its underlying mechanisms in treating anemia of chronic disease have not yet been investigated.

In the present study, ASP was extracted from the root of *A. sinensis,* and the effect of ASP against ACD was evaluated in a well-established rat model of ACD induced by the injection of complete Freund's adjuvant (CFA). The antihepcidin mechanisms of ASP related to inflammatory cytokines were investigated, and the potential target of ASP in BMP/SMAD signaling was explored *in vitro* and *in vivo*. These studies will improve our understanding of how ASP improves the ACD and will support the commercial application of ASP.

## 2. Materials and Methods

### 2.1. Materials and Reagents

Standard sugars were obtained from Sigma-Aldrich Chemical Company. Human hepatocellular carcinoma (HepG2) cells were obtained from the Tongji Medical College. Fetal bovine serum (FBS), DMEM medium, trypsin EDTA, penicillin, and streptomycin were obtained from Gibco (Grand Island, NY, USA). Anti-mouse *β*-actin antibody, horseradish peroxidase- (HRP-) conjugated anti-mouse, and anti-rabbit secondary antibodies were purchased from Sigma (St. Louis, MO, USA). Primary antibodies against STAT3, SMAD4, p-STAT3, p-SMAD1/5/8, Hepcidin, p-JAK2, SOCS3, BMP6, Id1, ferroportin, ferritin, IKK*α*, NF-*κ*B, p-I*κ*B*α*, Histone3.1, and GAPDH were obtained from Cell Signaling Technology (Danvers, MA, USA). The bicinchoninic acid (BCA) protein assay kit was purchased from Beyotime biotechnology (China). All the other reagents and chemicals were of analytical grade.

### 2.2. Plant Collection and Preparation of ASP

The dry roots of *A. sinensis* (Umbelliferae) were obtained from the Union Hospital and were collected from Minxian (Gansu province, China) in October 2013. Plant identification was performed by Professor Jinlan Ruan (Faculty of Pharmaceutical Science, Tongji Medical College of Huazhong University of Science and Technology, Wuhan, China) in accordance with the identification standard of the Pharmacopoeia of the People's Republic of China. ASP was isolated and purified as previously described [13]. Briefly, the sliced roots of AS (200 g) were boiled twice with 1 L of distilled water for 100 min each time. The acidic and alkaline proteins in the water extracts were removed by modulating the pH using Ca(OH)_2_ and 3 M H_2_SO_4_. Crude polysaccharide was obtained by adding an equal volume of ethanol. After repeated freeze dissolution and dialysis (3500 Da), purified ASP (the yield, 2.76%; the sugar content by the phenol-sulfuric acid method, 95.1%) was ultimately prepared through a gel filtration chromatograph with Sephadex G-50 and lyophilization. The determination of the associated chemical structures of ASP was performed by a variety of spectroscopic analyses as described previously [13].

### 2.3. Animals

The animal care and experimental procedures were carried out in accordance with *the Guidelines of the Institutional Animal Care and Use Committee of Tongji Medical College and the National Institutes of Health Guide for the Care and Use of Laboratory Animals* (permit number: SYXK (Hubei) 2010–0057). Male Sprague-Dawley rats (Animal Center of Tongji Medical College, Wuhan, China) were kept on a standard rodent diet until they reached an age of 6 weeks, and they weighed 160–180 g. The animals had free access to food and water and were housed according to the institutional and governmental guidelines in the animal facility of Huazhong University of Science and Technology with a 12-hour light-dark cycle and an average temperature of 20°C ± 1°C.

The rats were inoculated on day 0 with a subcutaneous injection of 0.15 mL of CFA (number 7027, Chondrex, Washington, USA), containing 10 mg/mL of mycobacteria into the paw of the left hind limb of each rat. The control rats received subcutaneous injections of sterile 0.85% saline. For the ASP experiments, the control rats and one group of the CFA-injected rats received an intragastrical administration of 1 mL of water (ACD) daily for 4 weeks. One group of the CFA-injected rats were treated intraperitoneally (IP) with erythropoiesis-stimulating agents (ESA; recombinant human erythropoietin, Sunshine Pharmaceutical Co., Shenyang, China) at a dose of 2000 U/kg body weight (BW) three times on three consecutive days, starting from day 25 after the CFA injection. Another cohort of CFA-injected rats were treated with intragastrical administrations of ASP at two dosages (0.5 (ASP1) or 1.0 (ASP2) g/kg BW), an intragastrical administration of Diclofenac sodium (5 mg/kg BW, Simcere, Jiangsu, China) alone (DF) or a combination of Diclofenac sodium and ESA (DF/ESA) for 28 days, starting from day 0 after the CFA injection.

After 4 weeks of treatment, the rats were anesthetized by an injection of 3% sodium pentobarbital (150 mg/kg), blood was collected from the tail veins and, then, the rats were euthanized. The livers and spleens were harvested for iron content, protein extraction, or immunohistology.

### 2.4. Hematological and Iron Analysis

To determine the hemoglobin (Hb) concentrations, red blood cell (RBC) counts and white blood cell (WBC) counts, blood, in ethylene diamine tetra-acetic acid- (EDTA-) containing vacuum tubes, was analyzed by an automatic blood counter (MEK-6318K, Nihon Kohden, Tokyo, Japan). The serum iron and spleen iron contents were determined with a flame atomic-absorption spectrometer (Model AA-240 FS, Varian Co., Palo Alto, CA, USA). The determination of the IL-6 and TNF-*α* concentrations was carried out using commercially available ELISA kits obtained from R&D Systems (Minneapolis, MN, USA). Serum hepcidin, ferritin, and erythropoietin levels were determined by an enzyme-linked immunosorbent assay (ELISA) according to the manufacturer's instructions (USCN Life Co., Houston, TX, USA).

### 2.5. Perl's Prussian Blue Staining

Spleen tissues were fixed in a 4% paraformaldehyde solution followed by a decalcification process for 2 weeks. After decalcification, the samples were processed, embedded in paraffin and sectioned. The slides of spleen tissues were stained with a Perl's Prussian blue solution (HT20-1KT, Sigma Aldrich, St. Louis, MO, USA) for 30 min at room temperature and were counterstained with neutral red based on a standard procedure for general evaluation. The slides were examined by an optical microscope (Nikon Eclipse Ci, Nikon Corporation, Tokyo, Japan).

### 2.6. Cell Culture and Treatments

Human hepatocellular carcinoma (HepG2) cells were cultured in DMEM medium supplemented with 10% FBS and 1% penicillin/streptomycin solution at 37°C in 5% CO_2_. The HepG2 cells were seeded onto six-well plates at 1 × 10^6^ cells per well. For the LPS treatment, the HepG2 cells were stimulated with 1 *μ*g/mL LPS (Sigma Aldrich, St. Louis, MO, USA) in the presence or absence of ASP for 24 hours and were then harvested for mRNA and protein analysis. The determination of the IL-6 concentration in the cell culture supernatants was carried out using a human IL-6 ELISA kit obtained from R&D Systems (Minneapolis, MN, USA). For the IL-6 treatment, the HepG2 cells were pretreated with ASP for 16 hours and were subsequently incubated with 50 ng/mL recombinant human IL-6 (PeproTech, Rocky Hill, Connecticut, USA) for another 8 hours. For the BMP6 treatment, the HepG2 cells were stimulated with 50 ng/mL BMP6 (PeproTech, Rocky Hill, Connecticut, USA) in the presence or absence of ASP (200 *μ*g/mL) for 24 hours. AZD1480 (5 *μ*M, Selleckchem S2162) and LDN-193189 (1 *μ*M, Selleckchem S2618) were prepared as DMSO solutions and were applied to the HepG2 cells 30 min before exposure to the stimuli. In addition, the same volume of DMSO was added in parallel as a vehicle control.

### 2.7. Quantitative Real-Time PCR

Total RNA was extracted from the HepG2 cells using TRIzol (Invitrogen, Carlsbad, CA, USA) according to the manufacturer's protocol. Complementary DNA was synthesized using the All-in-One™ First-Strand cDNA Synthesis kit (GeneCopoeia, USA). The quantitative reverse transcription polymerase chain reaction (qRT-PCR) was carried out in an ABI 7900 real-time PCR system (Illumina, San Diego, CA, USA) for 40 cycles using the All-in-One qPCR SYBR Green Mix (GeneCopoeia, USA). The primers were purchased commercially (Tsingke Biological Technology, Beijing, China). The sequences of the primers used were as follows: Hamp forward, 5′-CAACAGACGGGACAACTTGCA-3′, and reverse, 5′-AGTGGGTGTCTCGCCTCCTT-3′; *β*-actin forward, 5′- AGCGAGCATCCCCCAAAGTT-3′, and reverse, 5′-GGGCACGAAGGCTCATCATT-3′. The cycle threshold difference (ΔCt) values were obtained by subtracting the Hamp Ct values to the *β*-actin Ct (ΔCt = Ct_Hamp_−Ct_*β*-actin_). The results were expressed as the means of the fold changes in the Hamp gene expression relative to *β*-actin (2^−ΔΔCt^) ± SEM, *n* = 3.

### 2.8. Western Blotting

Nuclear extracts were prepared from LPS-induced HepG2 cells using a commercially available Nuclear/Cytosol Extract kit (Beyotime, Shanghai, China) and were used for a Western blotting analysis of NF-*κ*B expression. Total protein extracts were prepared from nitrogen-frozen rat livers or HepG2 cells in RIPA lysis buffer (50 mM Tris-HCI at pH 7.4150 mM NaCl, 1% Triton X-100, 1% sodium deoxycholate, and 0.1% SDS) containing PMSF, protease inhibitors, and phosphatase inhibitors (Beyotime, Shanghai, China) and were used for a Western blotting analysis of the other protein expressions. The protein content was determined by the bicinchoninic acid assay (BCA assay, Beyotime, Shanghai, China), and 80 *μ*g of total protein was separated by 10% SDS-PAGE and was transferred to a nitrocellulose membrane (NC, Amersham Pharmacia Biotech, England). The membranes were blocked for 2 h at room temperature with 5% skimmed milk or 5% BSA in TBST and then were probed with a STAT3 antibody, phospho-STAT3- (Tyr705-) antibody, SMAD4 antibody, phospho-JAK2 antibody, phospho-SMAD1/5/8 antibody, NF-*κ*B p65 antibody, phospho-I*κ*B*α* antibody, IKK*α* antibody, SOCS3 antibody, Id1 antibody, BMP6 antibody, ferroportin antibody, ferritin antibody, and hepcidin antibody. GAPDH or *β*-actin was used as a loading control. The membranes were then washed and incubated with horseradish peroxidase- (HRP-) conjugated anti-mouse or anti-rabbit secondary antibodies for 2 hours at room temperature. The membranes were washed again as above and were, then, developed with enhanced chemiluminescence (ECL, Thermo Scientific, Waltham, MA, USA) and visualized with the Gene Gnome XRQ (Syngene, UK). The protein levels were quantified by densitometry using the ImageJ program (version 1.48v, National Institutes of Health, USA).

### 2.9. Immunofluorescence Staining

The HepG2 cells were fixed with 4% formaldehyde and were treated with ice-cold methanol for 10 min at −20°C. After blocking in PBS with 5% BSA and 0.3% Triton X-100 for 1 hour at room temperature, the cells were incubated with the phospho-STAT3- (Tyr705-) antibody diluted in PBS with 1% BSA and 0.3% Triton X-100 at 4°C overnight. An Alexa Fluor 488-conjugated goat anti-rabbit IgG (Invitrogen, Carlsbad, CA, USA) was used as the secondary antibody, which was diluted in PBS with 0.1% Tween 20 and was incubated for 1 hour in the dark, followed by staining with DAPI for another 10 min. The slides were observed using a fluorescence microscope (Nikon E400, Nikon Corporation, Tokyo, Japan).

### 2.10. Statistical Analysis

The data are presented as the means ± standard error of mean (SEM). Comparisons between multiple groups in this study were performed by a one-way analysis of variance (ANOVA) with either LSD (assuming equal variances) or Dunnett's T3 (not assuming equal variances) for the post hoc analysis. A corrected *P* value less than 0.05 was regarded as statistically significant. All the data were analyzed with SPSS version 19.0 software (SPSS, Chicago, IL, USA).

## 3. Results

### 3.1. Chemical Structures of ASP

**Figure 1:**
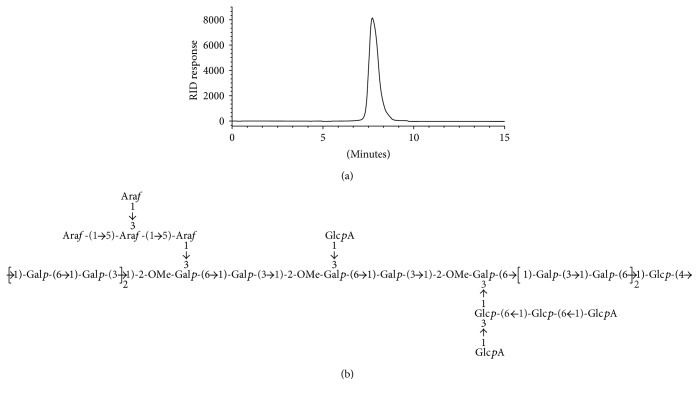
Characterization of ASP. (a) HPGPC-RID chromatogram of ASP. (b) Structure of the repeating unit of ASP used in this work.

A water-soluble acidic polysaccharide was extracted from the root of *A. sinensis*. The percentage of total carbohydrates was determined to be 95.5%. As shown in Figure 1(a), it exhibited a single and symmetrical peak in the HPGPC analysis, revealing that ASP was a homogeneous polysaccharide, and the molecular weight was determined as 80 kDa, according to the retention time. The main chains of ASP were composed of (1 → 3) linked galactopyranose (Gal*p*), (1 → 6) linked Gal*p*, and 2-OMe- (1 → 6) linked Gal*p*, which had three side chains attached to O-3 of 2-OMe- (1 → 6) linked Gal*p* and terminated with (1→) linked glucopyranuronic acid (Glc*p*A) and arabinofuranose (Ara*f*) (Figure 1(b)).

### 3.2. ASP Ameliorates Anemia and Increases EPO Production in Rats Suffering from ACD

**Figure 2:**
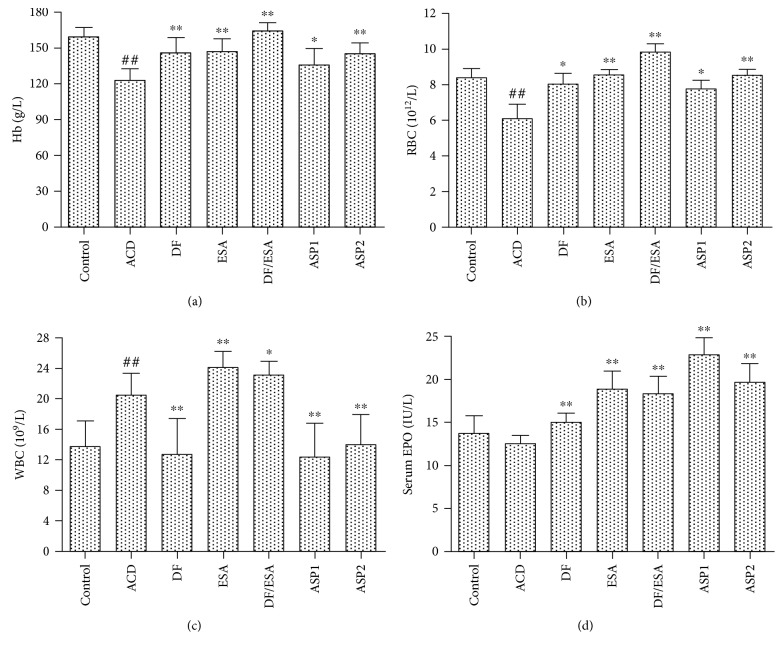
Effects of ASP on hematological parameters and serum EPO levels in rats. ACD rats were treated with either water (ACD), ASP (0.5 g/kg (ASP1); 1 g/kg (ASP2)), recombinant human erythropoietin (2000 U/kg, ESA), Diclofenac sodium alone (5 mg/kg, DF), or in combination with rhEPO (DF/ESA) over 28 days. (a) RBC counts, (b) Hb concentration, and (c) WBC counts were analyzed by an automatic blood counter. (d) Serum EPO was determined by ELISA. Results are expressed as means ± SD, *n* = 8/group. ^**##**^*P* < 0.01 versus control, ^∗^*P* < 0.05, ^∗∗^*P* < 0.01 versus ACD.

As shown in Figure 2, 28 days after CFA immunization, the mean Hb concentration and RBC counts of the ACD rats dropped by 36.38 g/L and 1.43 × 10^12^/L, respectively (*P* < 0.01 and *P* < 0.01), which showed severe anemia in the ACD rat group. The mean Hb concentration of the ASP-treated rats increased significantly compared to that of the ACD rats. Moreover, when ASP was used at a dose of 1.0 g/kg, the Hb concentration was statistically similar (*P* > 0.05) to the control rats, which was similar to the effect of ESA. Moreover, this effect was accompanied by enhanced RBC counts after ASP treatment compared with the untreated ACD rats (*P* < 0.05 and *P* < 0.01) (Figure 2(b)). In parallel, both the Diclofenac and ASP treatments resulted in a dramatic decrease in WBC counts compared to the ACD rats (Figure 2(c)). As revealed by these results, treatment with ASP effectively reversed inflammatory anemia in rats. The serum EPO levels were not significantly different in between the ACD rats and the control rats (Figure 2(d)). In contrast, ASP treatment (at both doses of 0.5 and 1.0 g/kg) stimulated EPO production to 22.85 ± 2.0 and 19.7 ± 2.1 U/L, respectively, which was stronger than ESA and Diclofenac.

### 3.3. ASP Treatment Decreases Inflammatory Cytokine Production by Suppressing NF-*κ*B Signaling

**Figure 3:**
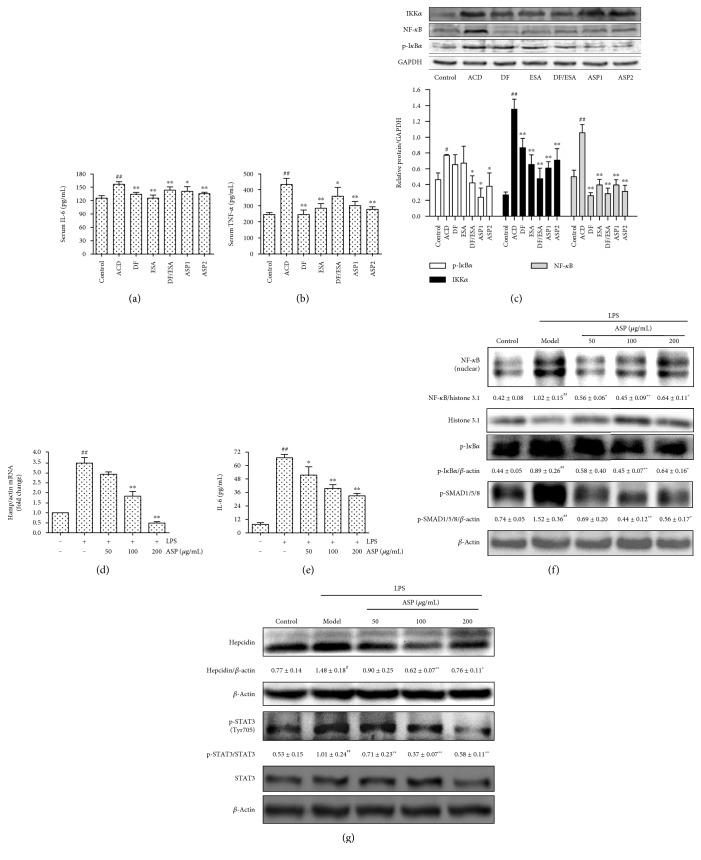
ASP treatment reduces proinflammatory cytokines levels and NF-*κ*B activation. (a) Serum IL-6 and serum (b) TNF-*α* levels were measured by ELISA. Data are reported as means ± SEM (*n* = 8 per group). ^#^*P* < 0.05, ^##^*P* < 0.01 versus control. ^#^*P* < 0.05, ^##^*P* < 0.01 versus control. ^∗^*P* < 0.05, ^∗∗^*P* < 0.01 versus ACD. (c) Immunoblots of protein expressions of IKK*α*, NF-*κ*B p65, and p-I*κ*B*α* in the livers of ACD rats. GAPDH served as the loading control. One representative blot of 3 independent experiments is shown. (d) The hepcidin mRNA levels were quantified by qRT-PCR. Values shown were the means of fold changes in Hamp gene expression relative to *β*-actin (2^−ΔΔCt^) ± SEM, *n* = 3. (e) ASP significantly reduced IL-6 secretion evoked by LPS (*n* = 3). Values are means ± SEM from 3 independent experiments. (f, g) ASP suppresses LPS-induced NF-*κ*B activation and hepcidin expression in HepG2 cells. Immunoblots of protein expressions of hepcidin, NF-*κ*B, p-I*κ*B*α*, STAT3, and p-STAT3. *β*-Actin and histone 3.1 served as the loading control. ^#^*P* < 0.05 and ^##^*P* < 0.01 compared with the control group; ^∗^*P* < 0.05 and ^∗∗^*P* < 0.01 compared with the LPS group.

Inflammation is involved in the mechanism of ACD, and therefore, we investigated the effect of ASP against inflammatory cytokines on ACD rats. Figures 3(a) and 3(b) shows that ASP treatment prominently reduced serum TNF-*α* and IL-6 levels. A Western blot demonstrated that ASP reduced NF-*κ*B p65 expression. To further explore the mechanisms of the ASP-reduced NF-*κ*B p65 activation, we analyzed the expressions of IKK*α* and phosphorylated I*κ*B*α*. Interestingly, the expressions of I*κ*B*α* phosphorylation and IKK*α* decreased significantly after ASP treatment.

To ascertain the involvement of NF-*κ*B inhibition by ASP in hepcidin expression, we treated the HepG2 cells with LPS, which activates NF-*κ*B via the Toll-like receptor 4- (TLR4-) associated signaling pathway in hepatocytes [14]. As shown in Figure 3(d), LPS increased hepcidin mRNA levels 3.5-fold, which was dose-dependently suppressed by ASP. In parallel, hepcidin protein expression induced by LPS was markedly reduced by the ASP treatment (Figure 3(g)). Moreover, the immunoblot assays indicated that ASP significantly inhibited p-I*κ*B*α* expression and the nuclear translocation of NF-*κ*B p65. We next analyzed the IL-6 content in the cell culture supernatants by ELISA. As presented in Figure 3(e), ASP dose-dependently ameliorated IL-6 secretion evoked by LPS. In agreement with the decrease in IL-6 levels, STAT3 phosphorylation was markedly inhibited by ASP.

#### 3.3.1. ASP Administration Inhibits Hepcidin Expression by Blocking JAK2/STAT3 and BMP/SMAD Signaling

**Figure 4:**
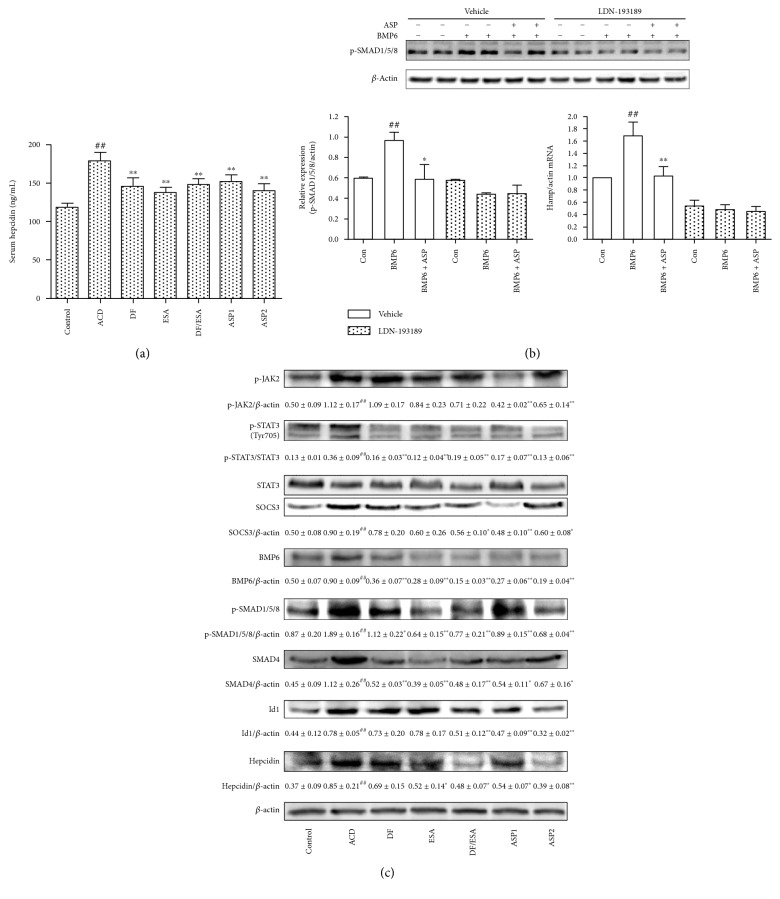
ASP suppresses hepcidin expression through blocking JAK2/STAT3 and BMP/SMAD signaling. ACD rats were treated with either water (ACD), ASP (0.5 g/kg (ASP1); 1 g/kg (ASP2)), recombinant human erythropoietin (2000 U/kg, ESA), Diclofenac sodium alone (5 mg/kg, DF), or in combination with rhEPO (DF/ESA) over 28 days. (a) Serum hepcidin levels were determined by ELISA. Values are reported as means ± SEM (*n* = 8 per group). (b) HepG2 cells were stimulated with 50 ng/mL BMP6 in the presence or absence of ASP (200 *μ*g/mL) and LDN-193189 (1 *μ*M) for 24 hours. P-SMAD1/5/8 expression was analyzed by Western blotting. The hepcidin mRNA levels were quantified by qRT-PCR. (c) Immunoblots of protein expressions of p-JAK2, p-STAT3, STAT3, SOCS3, BMP6, p-SMAD1/5/8, SMAD4, Id1, and hepcidin in the livers of ACD rats. *β*-Actin served as the loading control. One representative blot of 3 independent experiments is shown. ^#^*P* < 0.05, ^##^*P* < 0.01 versus control. ^∗^*P* < 0.05, ^∗∗^*P* < 0.01 versus ACD.

We next assessed the inhibitory effect of ASP on inflammation-induced hepcidin expression in ACD rats. As shown in Figures 4(a) and (c), treatment with ASP substantially reduced serum hepcidin levels and suppressed hepcidin expression in the liver compared with those in ACD rats, which was paralleled by the sole ESA treatment, the Diclofenac alone treatment, and the ESA/Diclofenac combined treatment.

To clarify the upstream signaling pathways regulating hepcidin expression, we then investigated the inflammation-inducible JAK2/STAT3 pathway in the rat liver. As shown in Figure 4(c), the expressions of p-JAK2 and p-STAT3 were largely increased in the ACD rats but were attenuated by ASP treatment (*P* < 0.01). A similar result was demonstrated for the expression of SOCS3, a central regulator of IL-6/STAT3 signaling. BMP6 and SMAD4 protein expressions were tremendously lowered after ASP treatment. Moreover, p-SMAD1/5/8 levels were notably increased in the ACD rats but were attenuated by ASP. Id1, an indicator of BMP/SMAD signaling activation, was strongly inhibited by ASP. Collectively, these results indicated that ASP suppressed hepcidin expression via reducing JAK2/STAT3 and BMP/SMAD activation in a rat model of ACD.

As BMP/SMAD signaling is the primary regulating pathway of hepcidin expression, we examined the effects of ASP on BMP6-mediated SMAD1/5/8 phosphorylation and hepcidin induction in HepG2 cells. As shown in Figure 4(b), p-SMAD1/5/8 and hepcidin mRNA levels were significantly increased after BMP6 challenge but were attenuated by ASP. To explore how ASP blocked BMP6-mediated SMAD1/5/8 phosphorylation and hepcidin induction, we treated HepG2 cells with LDN-193189, an inhibitor of the BMP type I receptor. The results demonstrated that LDN-193189 effectively blocked SMAD1/5/8 phosphorylation and hepcidin induction, and no significant changes were observed with the ASP treatment. Taken together, these results suggested that ASP inhibited SMAD1/5/8 phosphorylation and hepcidin activation induced by BMP6, and this effect might occur by inhibiting the binding of BMP6 to the BMP type I receptor.

#### 3.3.2. ASP Attenuates IL-6-Mediated STAT3 Phosphorylation and Hepcidin Induction

**Figure 5:**
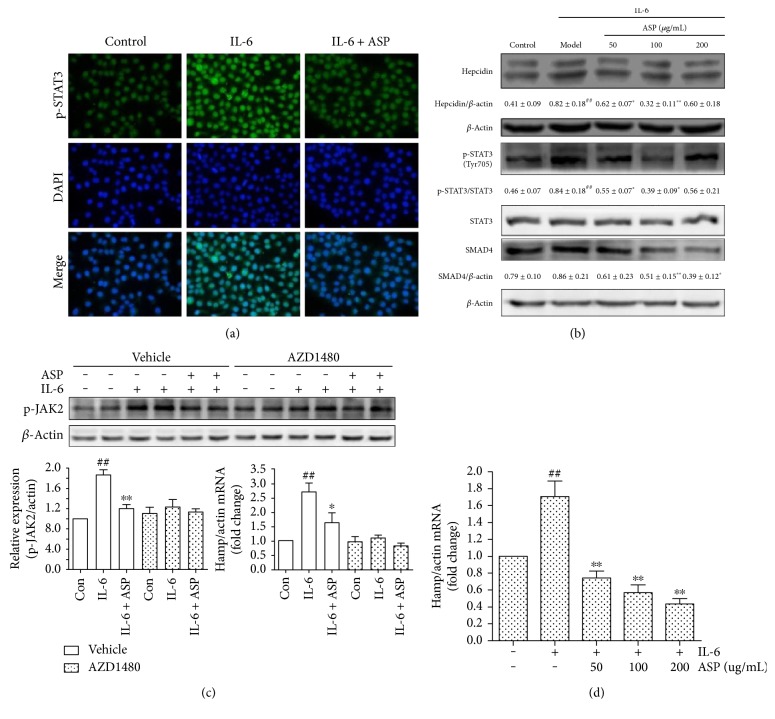
ASP inhibits IL-6-induced hepcidin expression and STAT3 phosphorylation as well as translocation in HepG2 cells. The cells were grown for 16 hours in the presence or absence of ASP, then added IL-6 (50 ng/mL) and grown for another 8 hours. (a) Representative immunofluorescence images illustrated that ASP successfully suppressed p-STAT3 nuclear translocation induced by IL-6.The hepcidin mRNA levels were quantified by qRT-PCR. (b) The STAT3 phosphorylation in response to IL-6, SMAD4, and hepcidin protein expressions was analyzed by Western blotting. One representative blot of 3 independent experiments is shown; *β*-actin served as the loading control. (c) HepG2 cells were pretreated with or without ASP (200 *μ*g/mL) for 16 hours and then stimulated with 50 ng/mL IL-6 in the presence or absence of AZD1480 (5 *μ*M) for another 8 hours. p-JAK2 expression was analyzed by Western blotting. (d) The hepcidin mRNA levels were quantified by qRT-PCR. Values shown were the means of fold changes in Hamp gene expression relative to *β*-actin (2^−ΔΔCt^) ± SEM, *n* = 3. ^#^*P* < 0.05 and ^##^*P* < 0.01 compared with the control group, ^∗^*P* < 0.05 and ^∗∗^*P* < 0.01 compared with the IL-6 group. Micrographs (magnification, 400x) are representative of one of three similar experiments and taken with Nikon E400.

As IL-6 is a primary driver for inflammation-inducible hepcidin expression in ACD [15], we next investigated whether ASP modified IL-6-induced hepcidin expression. IL-6 stimulation dramatically increased hepcidin mRNA and hepcidin protein expression. As presented in Figure 5(d), ASP dose-dependently decreased the level of hepcidin mRNA. It also reduced hepcidin protein expression and suppressed p-STAT3 induction by IL-6. Interestingly, the presence of ASP reduced SMAD4 expression, although SMAD signaling was not stimulated by IL-6. Immunofluorescence staining of p-STAT3 showed that ASP significantly decreased p-STAT3 nuclear translocation induced by IL-6 (Figure 5(a)).

To further explore the mechanisms by which ASP suppressed IL-6-induced STAT3 activation and hepcidin expression, we examined the effects of ASP on the activation of JAK2. As presented in Figure 5(c), ASP significantly reduced JAK2 phosphorylation. AZD 1480, an inhibitor of JAK2, effectively blocked JAK2 phosphorylation and hepcidin activation induced by IL-6. In AZD1480-treated cells, ASP did not significantly modulate JAK2 phosphorylation and hepcidin expression compared with the IL-6-treated group. These results suggested that ASP exerted an inhibitory effect on IL-6-induced STAT3 phosphorylation and hepcidin activation, possibly by inhibiting JAK2 activation.

### 3.4. Treatment with ASP Mobilizes Iron and Improves Hypoferremia in Rats Suffering from ACD

**Figure 6:**
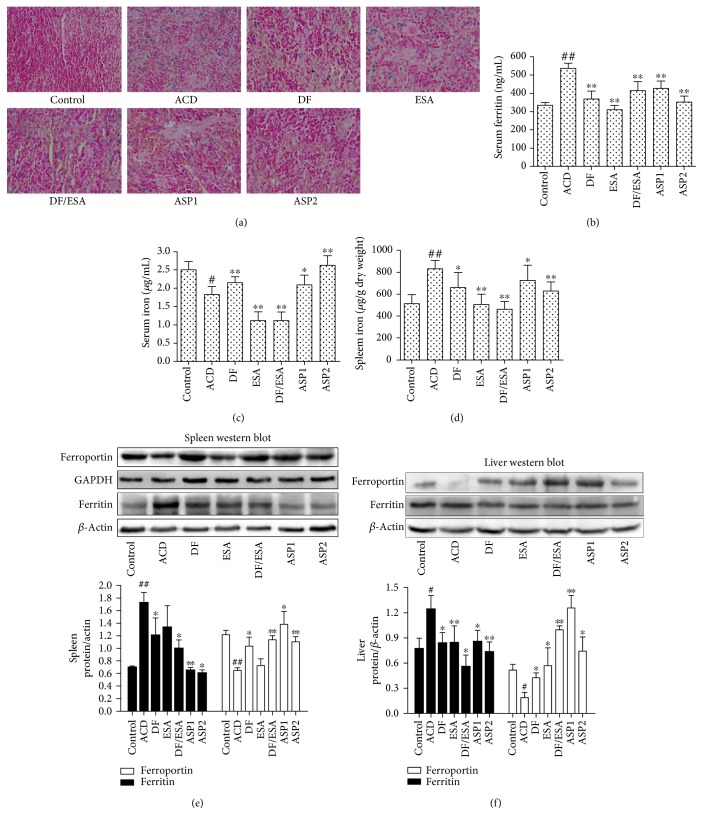
Effects of ASP treatment on iron homeostasis in ACD rats. (a) Prussian blue staining was performed on sections of formalin-fixed, paraffin-embedded spleens. (b) Serum ferritin levels were analyzed with a rat ferritin ELISA kit. (c) Serum iron and (d) spleen iron were measured spectrophotometrically. Immunoblots of protein expression of ferroportin and ferritin in the (e) spleens and (f) livers of ACD rats. *β*-Actin served as the loading control. One representative blot of 3 independent experiments is shown. Data are expressed as means ± SEM for 8 rats per group. ^#^*P* < 0.05, ^##^*P* < 0.01 versus control. ^∗^*P* < 0.05, ^∗∗^*P* < 0.01 versus ACD. Micrographs (magnification, 400x) are representative of one of three similar experiments and taken with Nikon Eclipse Ci.

The dysregulation of iron homeostasis is an important pathophysiological mechanism of ACD, and therefore, we next investigated the effect of ASP on iron metabolism. Prussian blue staining showed evident iron accumulation in the spleens of the ACD rats, and this iron retention was also ameliorated by ASP treatment (Figure 6(a)). Serum iron levels were markedly increased by both the ASP (*P* < 0.05, *P* < 0.01) and Diclofenac sodium (*P* < 0.01) treatment compared to the ACD rats, while the spleen iron levels were diminished (Figures 6(b), 6(c) and 6(d)). Immunoblot assays indicated that the expression of spleen ferroportin, which is decreased to prevent iron efflux into the plasma in ACD rats, was restored by ASP treatment (Figure 6(e)). Moreover, the level of iron storage protein ferritin in the spleen was markedly elevated in the ACD rats, and it declined after the ASP treatment. These changes in the spleen were paralleled by increased ferroportin levels in the liver and a reduced liver ferritin expression by the ASP treatment. In agreement with the reduction of ferritin levels in the spleen and liver, serum ferritin levels were markedly reduced with ASP administration. Taken together, these results demonstrated that ASP treatment counteracted iron retention within RES and improved hypoferremia.

## 4. Discussion

In the present study, we provided novel evidence for the effective treatment of ACD with a water-soluble acidic polysaccharide from *A. sinensis*, which not only suppressed inflammatory hepcidin but also reduced inflammatory cytokine secretion by inhibiting NF-*κ*B activation.

It is currently well characterized that the inflammatory induction of hepcidin expression is mainly mediated by IL-6, which binds to the IL-6 receptor and gp130 and then recruits Janus kinase 2 (JAK2) to phosphorylate STAT3 at tyrosine residue 705 [16, 17]. Furthermore, phosphorylated STAT3 translocates in the nucleus and binds the STAT3-responsive element in the hepcidin promoter, resulting in the transcription of hepcidin gene. Either liver-specific STAT3 knockout or IL-6 knockout mice demonstrate attenuated hepcidin induction or anemia in response to inflammatory stimuli [18, 19]. Consistently, ASP suppresses STAT3 phosphorylation and nuclear translocation as well as hepcidin induction under IL-6 stimulation, which is, at least partially, attributed to the inhibition of JAK2 activation. In parallel, treating ACD rats with ASP dramatically inhibited JAK2/STAT3 activation. Additionally, we observed that ASP reduced IL-6 secretion in both ACD rats and LPS-induced HepG2 cells, leading to decreased JAK2/STAT3 signaling and further contributing to hepcidin suppression.

BMP/SMAD signaling also contributes to hepcidin induction during inflammatory conditions, likely via activin B and SMAD-STAT3 interactions at the hepcidin promoter [20, 21]. BMPs bind to the BMP type II receptor and the BMP coreceptor hemojuvelin (HJV), which interact with the HFE/transferrin receptor-2 (TfR2) [22]. The interaction leads to the phosphorylation of the BMP-type I receptor, and phosphorylated type I receptor initiates the phosphorylation of the transcription factors SMAD1/5/8, which subsequently form a complex with SMAD4 and translocate to the nucleus to induce the transcription of the hepcidin gene [22, 23]. A recent report demonstrated that BMP6 directly activates SMAD1/5/8 signaling and induces hepcidin expression independent of the interaction with HJV/HFE/TfR2 [24]. Of note, the blockage of the BMP/SMAD pathway attenuates IL-6-induced hepcidin expression in both HepG2 cells and SMAD4-KO mice [25, 26]. In this work, we found that ASP blocked BMP6-mediated SMAD1/5/8 phosphorylation and Hamp induction and showed no response when the BMP-type I receptor was inhibited by LDN-193189. Consistent with the *in vitro* results, ASP decreased SMAD1/5/8 phosphorylation in a rat model of ACD. At the same time, BMP6 expression decreased after the ASP treatment, and this decrease might be associated with the reduction of intracellular iron levels. Thus, the suppression of BMP6 binding to the BMP-type I receptor by ASP might be a potential mechanism involved in the blockade of BMP/SMAD signaling.

Transferrin-bound iron in plasma is an exclusive source for erythropoiesis, and the majority of iron comes from the recycling of senescent erythrocytes by macrophages [27]. Ferroportin is the only known iron exporter that is responsible for the delivery of iron from macrophages of the spleen and liver and recycles senescent erythrocytes to plasma. Hepcidin controls the concentration of plasma iron by binding to ferroportin and then triggering ferroportin internalization and degradation. The degradation of ferroportin by hepcidin restricts the release of iron in splenic and hepatic macrophages and decreases plasma iron concentration, which results in iron-restricted erythropoiesis and anemia. In the rat model of ACD, we observed increased ferritin expression, both in the spleen and liver, and iron accumulation in the spleen along with reduced serum iron, indicating a diversion of iron from the circulation into the RES. However, we found highly elevated serum iron and markedly reduced serum ferritin after 4 weeks of ASP treatment. Additionally, the suppression of hepcidin expression by ASP substantially modulated iron transport from the spleen and liver as reflected by the increased ferroportin expression in both the spleen and liver, together with the reduced spleen iron and ferritin expression in the spleen and liver of the ACD rats. These results demonstrated that ASP inhibited inflammatory hepcidin expression to increase iron mobilization and restore the iron supply for hemoglobin synthesis.

The suppressive effect of inflammatory cytokines on erythropoiesis proceeds in concert with hepcidin-mediated iron retention in the development of ACD. The glycoprotein hormone EPO is an essential growth factor for erythroid progenitors, which binds to its receptor (EPOR) on hematopoietic progenitors to activate the downstream JAK2/STAT5, PI3K/AKT, and MAPK pathways, which, thus, promotes erythroid progenitor differentiation, survival, and proliferation [28]. The plasma level of EPO in patients suffering from ACD is often low in relation to the Hb level, and the enhanced inflammatory cytokines, mainly IL-1 and TNF-*α*, are responsible for the defect in EPO formation [29]. In this study, the results demonstrated that ASP treatment significantly reduced the production of the proinflammatory cytokines IL-6 and TNF-*α* in rats with ACD. Moreover, the treatment with ASP substantially increased serum EPO levels. Thus, it is possible that ASP increases EPO production, not only by reducing inflammatory cytokine production but also by stimulating endogenous erythropoietin secretion.

Most importantly, we showed here that increased iron mobilization, as well as reduced inflammatory cytokines, considerably increased hemoglobin and RBC levels after 4 weeks of ASP treatment. In the presence of the direct effect of inflammation on erythropoiesis, the iron consumption of erythropoiesis declines to avoid iron excess for the impaired erythroid machinery [30]. Rescuing suppressed erythropoiesis by anti-inflammatory interventions may potentiate the therapeutic efficacy of antihepcidin interventions aimed at enhancing iron availability. Altogether, we demonstrated that a natural polysaccharide was effective at reversing anemia in ACD, not only by inhibiting hepcidin expression but also by lowering inflammatory cytokine production, with subsequent increased iron availability and a rescue of suppressed erythropoiesis.

We then explored the molecular mechanism of the ASP anti-inflammatory effect. NF-*κ*B plays a key role in regulating proinflammatory gene expression, and it induces the transcription of proinflammatory cytokines, including TNF-*α*, IL-1*β*, and IL-6 [31]. NF-*κ*B activation is regulated by inhibitors of *κ*B (I*κ*B) and I*κ*B kinases (IKKs), whereby activated IKK initiates I*κ*B*α* phosphorylation [32]. Phosphorylated I*κ*B*α* is then ubiquitinated and, subsequently, is degraded by the proteasome, thereby allowing NF-*κ*B proteins to translocate to the nucleus and induce the transcription of proinflammatory genes [32]. In the present study, ASP treatment resulted in a significant reduction of IKK*α* and phosphorylated I*κ*B*α* expressions and, subsequently, inhibited NF-*κ*B p65 activation in rats suffering from ACD. Similarly, we observed that ASP suppressed the phosphorylation of I*κ*B*α* and then prevented the nuclear translocation of NF-*κ*B in LPS-induced HepG2 cells. Accordingly, the suppression of NF-*κ*B p65 activation by ASP is a potential mechanism involved in inflammatory cytokine reduction, which further promotes the antihepcidin effects of ASP.

**Figure 7:**
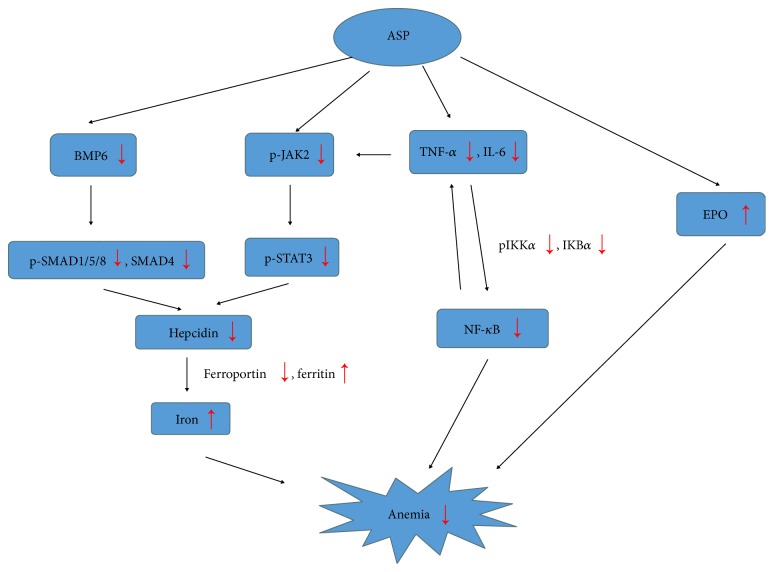
Flow diagram of the mechanism of ASP-reversed anemia of chronic disease.

In summary, we demonstrated that ASP was an efficient inhibitor of hepcidin expression in both HepG2 cells and in a rat model of ACD by blocking the BMP/SMAD and IL-6/STAT3 pathways. The administration of ASP improved iron availability, abrogated the suppression of erythropoiesis by inflammatory cytokines, and effectively reversed anemia via inhibiting inflammatory hepcidin and NF-*κ*B activation in ACD rats (Figure 7). Our work offers valuable results for natural ASP as a potential treatment option or complementary drug for patients suffering from ACD.
